# Neonatal Programming of Microbiota Composition: A Plausible Idea That Is Not Supported by the Evidence

**DOI:** 10.3389/fmicb.2022.825942

**Published:** 2022-06-17

**Authors:** Catherine Michel, Hervé M. Blottière

**Affiliations:** ^1^Nantes Université, INRAE, UMR 1280, PhAN, Nantes, France; ^2^Université Paris-Saclay, INRAE, MetaGenoPolis, Jouy-en-Josas, France

**Keywords:** neonatal gut microbiome, DOHaD, first thousand days of life, perinatality, programming

## Abstract

Underpinning the theory “developmental origins of health and disease” (DOHaD), evidence is accumulating to suggest that the risks of adult disease are in part programmed by exposure to environmental factors during the highly plastic “first 1,000 days of life” period. An elucidation of the mechanisms involved in this programming is challenging as it would help developing new strategies to promote adult health. The intestinal microbiome is proposed as a long-lasting memory of the neonatal environment. This proposal is supported by indisputable findings such as the concomitance of microbiota assembly and the first 1,000-day period, the influence of perinatal conditions on microbiota composition, and the impact of microbiota composition on host physiology, and is based on the widely held but unconfirmed view that the microbiota is long-lastingly shaped early in life. In this review, we examine the plausibility of the gut microbiota being programmed by the neonatal environment and evaluate the evidence for its validity. We highlight that the capacity of the pioneer bacteria to control the implantation of subsequent bacteria is supported by both theoretical principles and statistical associations, but remains to be demonstrated experimentally. In addition, our critical review of the literature on the long-term repercussions of selected neonatal modulations of the gut microbiota indicates that sustained programming of the microbiota composition by neonatal events is unlikely. This does not exclude the microbiota having a role in DOHaD due to a possible interaction with tissue and organ development during the critical windows of neonatal life.

## Introduction

The “developmental origin of health and disease” (DOHaD) theory that emerged a few decades ago (see Gluckman et al., 2016 for historical review) maintains that the state of health and risk of disease in adulthood are programmed by exposure to environmental factors during critical windows in early life, while tissues and organs are developing. Since it was first established, as a result of epidemiological studies that highlighted the relationship between birth weight and the subsequent risk of cognitive dysfunction, obesity and cardiovascular disease (e.g., Barker and Osmond, 1986), its scope has expanded considerably and now encompasses numerous non-communicable pathologies, as well as mental health (for review, see Hoffman et al., 2017). The notion of early life has also been discussed and refined, culminating in the “first 1,000 days of life” concept that is currently in use, referring to the period between conception and the end of the child’s second year (Darmaun, 2020).

The DOHaD theory has considerable implications for public health as it provides an opportunity for a strategy for preventing non-communicable diseases, which would consist of optimizing early nutrition and development to decrease the spread of these diseases and improve “human capital,” halting the intergenerational transmission of disease risks. In this respect, it is a major challenge to elucidate the mechanisms by which the “memory” of the early life environment is retained in later life and is responsible for this “programming.” So far, it has been mainly attributed to epigenetic modifications (for review, see Fall and Kumaran, 2019), but the involvement of the intestinal microbiome has also been proposed (e.g., Stewart and Cummings, 2017; Darmaun, 2020; Stinson, 2020).

This assumption results from a combination of the following well-known findings: (1) intestinal microbiota assembly coincides in part with the neonatal segment of the 1,000-day window (Doré and Blottière, 2015; Kennedy et al., 2021); (2) the initial assembly of bacteria is highly sensitive to the perinatal environment (e.g., Penders et al., 2006; Tun et al., 2017); and (3) depending on its composition and activity, the intestinal microbiome has a profound effect on host physiology far beyond the digestive sphere (for review, see van de Guchte et al., 2018). Specifically, the microbiota could contribute to DOHaD either as a relay: changes in the microbiota induced by the neonatal environment would affect its ability to interact with tissue and organ development during the critical windows of neonatal life with lasting consequences, or as a memory link between early environment and adult health: the initial bacterial configuration would shape the composition of the microbiome for life and therefore program its contribution to the regulation of host physiology. The first option has been the subject of several recent reviews (Stinson, 2020; Ríos-Covian et al., 2021, Sarkar et al., 2021) and will therefore be out of our scope. Conversely, the widely propagated assumption that early modifications of the neonatal microbiome program the microbiome throughout the entire life remains actually to be established.

To this end, this mini-narrative review summarizes the arguments supporting the plausibility of the gut microbiota being programmed by the neonatal environment and critically reviews the literature on monitoring the long-term effects of selected neonatal modulations of the gut microbiota, in both animals and humans. These two elements will help clarify the potential role of the gut microbiota in programming host health.

## The Early Gut Microbiota Assembly Would Determine Its Subsequent Composition

The intestine of a newborn, sterile *in utero* (Kennedy et al., 2021), is first colonized on birth by bacteria of maternal origin (from the vagina, the skin, and more particularly the feces) and/or from the environment (Bäckhed et al., 2015; Milani et al., 2017; Ferretti et al., 2018). From these pioneer bacteria, to which others are added through maternal milk (Laursen et al., 2021), a microbiota is formed, the density of which reaches a population level comparable to that of the adult (Doré and Blottière, 2015) within a few days. In the first months of life, the gut microbiome is chaotic and highly dynamic, and appears to be driven essentially by stochastic processes (Stewart and Cummings, 2017); its composition then gradually complexifies, progressing over time from a state of low diversity to form richer multispecies communities. Finally, the microbiome undergoes an increase in intra-individual diversity (alpha diversity) and a decrease in inter-individual diversity (beta diversity) (Vallès et al., 2014; Laursen et al., 2021), and achieves an adult-like community. In recent years, the age at which this adult composition is reached has been revised upward (approximately 6 years vs. 2–3 years previously) and is still under debate (Korpela and de Vos, 2018; Zhong et al., 2019; Roswall et al., 2021). In summary, the development of the microbiota follows trajectories (White et al., 2013; Roswall et al., 2021) shaped by ecological forces that remain poorly understood.

### Rationale for Priority Effects

The idea that colonization history, i.e., the order and timing of previous species settlement, is of crucial importance in the final assembly of the gut microbiota is widely shared in the literature. These priority effects were first supported by Eggesbø et al. (2011), who demonstrated statistical associations between specific microbiotal taxons detected in newborns aged 4 days and the abundance of other specific microbial groups at day 120, suggesting that early settlers may influence the subsequent composition of the microbiota. It was subsequently established by a checkerboard analysis of 60 fecal samples from a healthy, full-term child between birth and 2.5 years of age that the gut microbiome assembly is not random (Koenig et al., 2011). Similarly, after modeling the order in which new species are detected in microbial communities over time, Darcy et al. (2020) concluded that assembly during primary succession of the infant gut follows a predictable pattern since less new diversity is added than expected by chance. In line with this predictability, some authors succeeded in computing the age of gut microbiota using the abundance of certain taxa identified as indicators of a child’s age (Bäckhed et al., 2015; Xiao et al., 2021). Finally, the fact that the same evolutionary trajectories of microbiota composition between different clusters or enterotypes are shared by different individuals reinforces the idea that the transition of gut microbiota from infant to adult follows a deterministic pattern, which is further supported by the decrease in stochasticity ratios within the dominant enterotypes with age (Xiao et al., 2021).

### Potential Mechanisms for Priority Effects

These priority effects are underpinned by a theoretical framework that, as well as niche pre-emption monopolization, relies mainly on bacterial ability to modulate the intestinal environment and establish metabolic interactions, thus producing either favorable or inhibiting conditions for the establishment of newly encountered bacteria (Sprockett et al., 2018; Laursen et al., 2021). A classic example is that of reducing oxygen levels by primo-colonizing bacteria (facultative anaerobes) and consequently the creation of an environment favorable to the strictly anaerobic bacteria that implant later. This notion, however, has now been challenged since the presence of strict anaerobes has been reported very soon after birth, and no real difference is observed in the intestinal O_2_ content between axenic and conventional rodents (Bittinger et al., 2020). Other obvious examples include the luminal pH, which is perceived as a crucial abiotic factor in controlling newcomer settlement due to differences in the acid tolerance capability of different bacterial strains, and the difference in pKa of the various organic acids (e.g., lactic acid vs. acetic acid) produced by the bacteria during fermentation. These organic acids can also affect the intestinal motility and thus the turnover of bacterial populations through proliferation and fecal excretion. Together with pH, they have been identified as pivotal elements for bacterial cross-feeding and controlling microbiome stability (Wang et al., 2020). The existence of statistical associations between bacterial populations at various stages of development (Bergström et al., 2014) supports the involvement of these trophic interactions in the initial assembly of the intestinal microbiome. However, their precise contribution must be qualified: according to Laursen et al. (2021), they would explain the priority effects mainly in species that are more sensitive to specific requirements, while for Guittar et al. (2019), who studied microbiome functionality maturation, these traits had little influence on compositional variability over succession. Interactions other than trophic ones may also be involved, e.g., the production of bacteriocins by some bacterial species. In this respect, Morelli (2008) suggested that the reduced presence of clostridia in breastfed babies may arise from the presence of bacteria of the *Ruminococcus* genus, which is known to produce antimicrobial peptides (ruminococcins) that may inhibit the development of many species of *Clostridium* sp. It is also important to keep in mind that the evolution of the microbiota takes place at the same time as the intestinal maturation process, which affects both the immune and epithelial components of the colon, both of which are involved in the control of bacteria engraftment (Biol-N’garagba and Louisot, 2003; Zhang et al., 2015). As intestinal maturation can be reversibly influenced by the presence of bacteria (for reviews, see Ríos-Covian et al., 2021; Sarkar et al., 2021), its modulation by early settlers may – beyond constituting mechanisms for the possible role of early microbiota perturbations as a relay for later physiological consequences (not discussed here) – as well contribute to priority effects. Note that the maturation of these two tissues is otherwise paramount for the microbiome differences associated with newborn gestational age. The long-term determination of the microbiota in this case goes far beyond the issue of ecological forces alone, and therefore the differences between term and preterm babies do not fall within our scope here.

### Preclinical Evaluation of Priority Effects

The successional process through which the gut microbiome develops during infancy has also been investigated experimentally in two preclinical studies, which led to contradictory conclusions.

Martínez et al. (2018) demonstrated a historical contingency by comparing microbiota obtained from mice fed a standard rodent diet and conventionalized by combinations of inocula (different complex adult microbiota and specific strains) administered in varying order during neonatal development (inoculation on days 10 and 36 of life). In this study, arrival order had an influence on successful colonization for some species, the communities of the recipients were more similar to the donor community that arrived first, and the ability of certain pure strains to modify the overall structure of the microbiota was only expressed if administered first. In addition, by repeating the study in mice lacking adaptive immunity (Rag1-/-), the authors demonstrated that this historical contingency does not rely on education/priming of the adaptative immune response, a finding that does not preclude about a possible lasting influence on the innate response. Conversely, Feng et al. (2020), who conducted a similar experiment in older mice (3–4 weeks old) fed an infant formula, concluded that priority effects were not deterministic in their model. In this case, the inocula consisted of two consortia composed of intestinal bacterial strains isolated from a single infant and selected on the basis of their taxonomic relationships with operational taxonomic units (OTUs), which were statistically associated with the first two developmental stages of the microbiome (S1 and S2), as determined in a preliminary step. A comparison of the groups of animals subjected to different exposure timelines to the S1 consortium (comprising 21 bacteria) and S2 consortium (13 bacteria) revealed that bacteria from the S2 consortium (i.e., the later natural colonizers) generally outcompeted most of those in the S1 consortium regardless of the order of arrival. It remains to be seen whether these contrasting findings stem from differences in protocol (with particular respect to the age of the recipient mice and donors as well as to the diet), whether complete microbiomes or non-cultivable bacteria are used, or a combination of all these factors.

Another explanation could be the fact that these works focused solely on bacterial interaction and did not take into account the priority effects that are also possible between various types of microorganism within the broadly defined microbial community (Sprockett et al., 2018). In this respect, the evidence produced by Rao et al. (2021) supporting species-specific cross-kingdom interaction shaping the dynamic of microbes both *in vitro* and within the mouse gut is of particular interest. When measuring microbial colonization dynamics in specific-pathogen-free (SPF) mice pre-colonized with *Candida albicans, Candida parapsilosis*, or a control vehicle prior to inoculation with *Klebsiella pneumoniae*, these authors observed significantly reduced *K. pneumoniae* colonization in the mice pre-colonized with *C. albicans*, compared to those in the control group and those pre-colonized with *C. parapsilosis*. This would deserve consideration as fungal communities have been reported in the infant gut (Ward et al., 2017; Boutin et al., 2021). In the same vein, the broadly defined microbial community also includes a bacteriophage virome that fluctuates in early life (Lim et al., 2015; Shamash and Maurice, 2021). Xiao et al. (2021) found that changes in bacterial abundances within enterotypes were associated with changes in the abundance of their corresponding bacteriophages, suggesting that bacteriophages may be involved in the transition between enterotypes. This involvement may not be limited to childhood; in a study describing the main impacts of lifestyle on adult gut microbiota, Jha et al. (2018) identified “drinking water source” as one of the factors significantly associated with changes in the gut microbiome, which could be related to the role of environmental bacteriophages as microbiota structuring agents.

## Evidence of Microbiota Programming by the Neonatal Environment Is Missing

As described above, the assembly of the gut microbiome is sensitive to various perinatal events; the modulating factors and their impacts have both been described in numerous reviews (Mackie et al., 1999; Penders et al., 2006; Adlerberth and Wold, 2009; Fouhy et al., 2012; Edwards, 2017; Milani et al., 2017; Tun et al., 2017; Vandenplas et al., 2020; Stinson, 2020). The most historically documented modulating factors include mode of delivery [vaginal delivery vs. cesarean section (CS)] and method of early feeding (breast milk vs. formula). In these two cases, the impacts must be qualified: in terms of CS, the repercussions depend on whether it was a scheduled or emergency operation (e.g., Mitchell et al., 2020) while the impact of early feeding mode must take into account the duration of breastfeeding and type of infant formula [e.g., supplemented or not with lactoferrin, prebiotics, or human milk oligosaccharides (HMO), which may all affect bacterial growth] (e.g., Galazzo et al., 2020). Many other driving forces can also affect early microbiota composition, including the infant’s genetic background, antibiotherapies, gestational age at birth, place of birth, geographic location and older siblings, and also the state of hygiene of the environment, exposure to furry household pets, etc. Prenatal features such as maternal diet, obesity, smoking, and the use of antibiotic agents during pregnancy have also been identified as factors that vary the initial constitution of the child’s microbiota.

For the most instrumental of these factors (mode of birth, type of feeding, antibiotic therapy, genetics), few studies have considered the question of whether or not their impacts have long-lasting repercussions on the composition of the microbiome. Unfortunately, no longitudinal human studies over extended periods (i.e., several decades) and based on comparable methodological approaches have yet been carried out. To shed light on this issue, therefore, we also considered the contribution of animal studies that should reveal the potential for microbiota programming with a control of possible confounding factors.

### Mode of Delivery

Most studies addressing the lasting effects of the impact that delivery mode has on the gut microbiota agree that it gradually diminishes as the newborn matures, but there is no agreement as to the age at which this fading occurs and whether or not the observed normalization is complete.

Some authors argue for a rapid disappearance (i.e., less than 3 months) of the impact (Stewart and Cummings, 2017; Reyman et al., 2019) due to masking by other effects such as that of diet, particularly the cessation of breastfeeding (Galazzo et al., 2020). Rutayisire et al. (2016) and Korpela and de Vos (2018) concluded that the differences between infants born *via* vaginal delivery (VD) and those born by CS disappear by the end of the first year. Conversely, some studies show significant differences in the structure of the gut microbiota and/or its functional potential between these two types of infant up until the age of 1 year (Busi et al., 2021; Mueller et al., 2021) or 2 years, although markedly attenuated in the latter case (Podlesny and Fricke, 2021) and poorly translated into metabolic differences (Edwards, 2017). Only a few studies have considered the issue beyond this age. The oldest age was considered by Salminen et al. (2004), who, using a method targeting specific bacterial populations (FISH), reported a significantly reduced abundance of clostridia (as then defined) in the feces of children aged 7 years born by CS. Also using a targeted method (RT-qPCR), Nagpal and Yamashiro (2018) reported that although the differences they observed in carriage of some lactobacilli and bifidobacteria between CS and VD until the age of 6 months were no longer detected at 3 years of age (no intermediate time studied), they observed in an unpublished adult study that the subjects in the caesarean group had a significantly lower carriage rate for the *Bacteroides fragilis* group and *Lactobacillus sakei* subgroup compared to those in the VD group. The authors point out that these differences in carriage were not related to differences in abundance, thus raising the question of which characteristic of the microbiota should be taken into account when deciding on the durability of an impact. In this context, it is noteworthy that Fouhy et al. (2019) managed to identify a few bacterial taxons as characteristic of microbiota from infants born vaginally by Lefse Linear discriminant analysis effect size (LEfSe) when comparing CS and VD at 4 years, but failed to demonstrate any clustering of samples based on delivery mode. Similarly, Roswall et al. (2021) concluded that the impact of mode of birth on overall microbiome structure normalizes as the gut microbiota matures, but still detected significant differences in abundance for a few genera between children born by CS and those born vaginally, at the age of 5.

Similar disparity of conclusion depending on the nature of the microbiota characteristics analyzed is also found in one of the few studies carried out in animals and appropriately monitored with regard to the potentially confounding impact of fostering subsequent to CS. Birth delivery mode was shown to alter the microbiome structure in mice at 5 weeks of age, whatever the biodiversity metric used (Zachariassen et al., 2019). However, for older mice (8 weeks), this was only true for one of the metrics used (weighted UniFrac).

Altogether, these latter results suggest that, where they exist, long-lasting alterations in microbiota related to mode of delivery are minor.

### Antibiotics

It is difficult to draw any clear-cut conclusion on the long-lasting impact of early antibiotic treatment in humans, considering the enormous variations in prescriptions [type, dosage and possible combination of antibiotic(s), route and length of administration, indication for the prescription, culture/habit of the clinician] (Stewart and Cummings, 2017; Aires, 2021) and the specificities of the target population (intrapartum or neonatal administration, term or preterm neonates, vaginal or CS delivery) (Dierikx et al., 2020). The picture is further complicated by the possible cumulative nature of antibiotic treatments during neonatal development (Blaser, 2016). However, according to recent reviews (Azad et al., 2017; Edwards, 2017; Dierikx et al., 2020; Aires, 2021), the lasting impacts of perinatal antibiotherapies may be noticeable for a few months but not beyond, depending on the nature of the antibiotics (ATB) and possibly the age at which it was administered. Studies on durations longer than a few months are nevertheless rare. They include a study by Yassour et al. (2016), who reported detectable differences at the age of 3 years, but in this case the children included in the “antibiotic treatment” group had received between 9 and 15 treatments over the first 36 months of life.

The importance of the nature of the antibiotic and its administration protocol has been highlighted in preclinical studies, to which the Blaser teams made a major contribution (Cox et al., 2014; Nobel et al., 2015; Ruiz et al., 2017). With regard to amoxicillin (β-lactam antibiotic), we have described that when administered orally to lactated rat pups, its impact, although massive, disappeared very quickly once exposure was terminated (Morel et al., 2013). Both Nobel et al. (2015) and Li et al. (2017) reported a broadly similar conclusion with mice subjected to repeated amoxicillin pulses in postnatal days 10–40 and with piglets given continuous antibiotic treatment from birth to postnatal day 14. With regard to penicillin (another β-lactam antibiotic), the two studies carried out in mice based on perinatal administration suggest that the effects on the microbiota could last for some time (Leclercq et al., 2017) but would also eventually diminish (Cox et al., 2014). Conversely, the administration of macrolides (tylosin, tulathromycin) seems to induce long-lasting effects both when administered repeatedly during perinatal development in mice (Nobel et al., 2015; Ruiz et al., 2017) and as a single dose on the fourth day of life in piglets (Schokker et al., 2015).

As it is not macrolides but rather aminoglycosides (gentamycin) and penicillins (benzylpenicillin, ampicillin, amoxicillin, piperacillin) that are most frequently prescribed in neonatology worldwide (Krzyżaniak et al., 2016; Al-Turkait et al., 2020), there is therefore an overall consistency between preclinical and epidemiological findings.

### Infant Nutrition/Feeding Mode

Early nutrition is a crucial factor in controlling the early microbiota in terms of both its composition and its ability to produce metabolites. Historically, breastfeeding is universally accepted as being particularly impacting among influencing neonatal nutritional characteristics, but more recently the supplementation of infant formulas, with prebiotics and/or probiotics in particular, also contributes to the strategies to modulate the neonatal gut microbiome (Milani et al., 2017).

Whether the differences produced by breastfeeding status persist or not is not clearly defined. Some authors conclude that the differences last for a certain length of time: until school age for Zhong et al. (2019) and until 6 years of age according to Gschwendtner et al. (2019), while others consider that they disappear with food diversification and do not last beyond the first 24 months (Stark and Lee, 1982; Bazanella et al., 2017; Stewart and Cummings, 2017; Stewart et al., 2018). As demonstrated first by Roger and McCartney (2010) in a longitudinal follow-up of two infants, and subsequently confirmed by others (Bäckhed et al., 2015; Galazzo et al., 2020), the composition of the microbiota seems to change as soon as breastfeeding ceases and its diversity at 1 year is positively correlated with breastfeeding exclusivity and duration (Azad et al., 2016).

The persistence of the effects of breastfeeding beyond complete weaning seems to be confirmed by the few available animal studies conducted either in rats reared in cups (Nakayama et al., 2003), piglets (Brink et al., 2019; Elolimy et al., 2020), or rhesus macaques (Ardeshir et al., 2014). However, this should be viewed with caution due to possible biases related to the stresses induced in such studies (maternal separation and nutritional adequacy of the milk administered).

With regard to formula supplemented with prebiotics or probiotics, very few clinical trials have investigated whether their impacts on fecal microbiota persist long after they are discontinued (Edwards, 2017).

For prebiotics, significant microbiotal changes have been reported for post-supplement durations of between 2 weeks (Waligora-Dupriet et al., 2007) and 6 months (Salvini et al., 2011), with no information on further progression. Longer-lasting impact appears unlikely since differences in the relative abundance of the bifidobacteria, which are characteristic of prebiotic supplementation, are only detected at 2 and 6 months in infants supplemented for 12 months (Neumer et al., 2021). For probiotics, no systematic impact on microbiota composition has been observed (e.g., Rinne et al., 2006 vs. Bazanella et al., 2017). Where probiotics have effectively modulated the microbiota, studies have mainly focused on short-term persistence; differences were still observed 18 months after treatment was suspended in infants exposed to *Lactobacillus rhamnosus* GG from before birth until the age of 6 months (Rinne et al., 2006), whereas no significant difference in taxa abundance was detected after a 1-month washout period between infants fed a formula supplemented with *Bifidobacterium animalis* ssp. *lactis* CNCMI-3446 and others who were not (Castanet et al., 2020). Similarly, a brief characterization of the microbiome (by qPCR) revealed no significant differences in the gut bacterial composition of 3-year-old children whether they had been fed a formula supplemented with galacto-oligosaccharides (GOS) and *Lactobacillus fermentum* CECT5716 or with GOS only, between the first and sixth months of life (Maldonado-Lobón et al., 2015). Such discrepancies may arise from differences in the nature of the prebiotic or probiotic strains used and the dosages or time windows in which they are administered (Davis et al., 2020).

**FIGURE 1:**
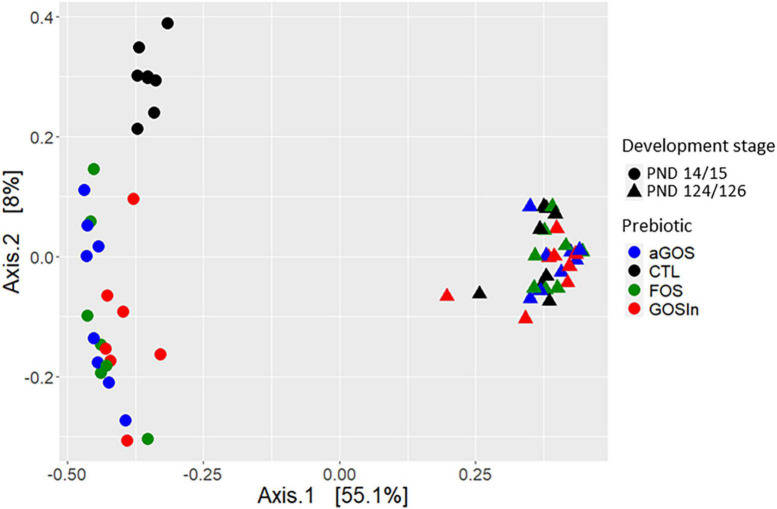
Illustration for the disappearance of the impact of neonatal prebiotic supplementations on caecal bacterial communities in rats: pups supplemented with different prebiotics [alpha Galacto-oligosaccharide, Fructo-oligosaccharides or a mix (9:1) of beta-Galacto-oligosaccharides and inulin] from post natal day 5–14/15 clustered together but did not overlap with pups from the CTL group at the end of the supplementation (PND14/15): they exhibited a clearly distinct microbial community as compared to unsupplemented pups. This was no longer true in adulthood (PND124/126) where all individuals clustered together [multidimensional scaling (MDS) analysis based on dissimilarities measures (Bray–Curtis) calculated from data published by Le Dréan et al., 2019].

For similar reasons or depending on the specific animal species or specific microbiota used in two experiments, animal studies have also generated non-uniform conclusions. In one of our study (Morel et al., 2015), a slight programming of the adult microbiota of rats was observed by pre-weaning supplementation with a particular mix of GOS and inulin (GOS/In). Concurring with this, Tian et al. (2019) demonstrated that changes in the composition and activity of the ileal microbiota in newborn piglets supplemented with GOS/In persisted 2 weeks after the end of the treatment, although they were diminished compared to the changes observed after 8 days. Conversely, in our second study using rats, none of the prebiotics (including GOS/In) administered before weaning induced any significant difference in the microbiota when the animals matured into adults, despite marked early impacts (Figure 1; Le Dréan et al., 2019). Finally, other studies using a prebiotic that had no programming impact in our own studies observed that supplementation with perinatal fructo-oligosaccharides induced persistent modulation of the composition of the adult (9 months) fecal microbiota in pigs (Le Bourgot et al., 2019). Given the disparity of the underlying protocols, these few examples are insufficient to draw any clear-cut conclusion as to the ability of neonatal prebiotic supplementation to induce microbiota programming. The dissimilarity of conclusions suggests, however, that this capacity, if it exists, is not considerable.

### Genetics

An impact on gut microbiome composition by the host genetics has been proposed following adult studies (based on studies on twins or relatives, or on genome-wide association studies; for reviews, see Spor et al., 2011; Campbell et al., 2012; Bubier et al., 2021), suggesting an enduring influence. However, the impacts of ABO antigen and secretory phenotypes initially described from targeted microbiota characterizations in small cohorts were not confirmed in a study based on 16S sequencing of fecal microbiota from a much larger cohort that considered the overall structure of the microbiome, its diversity, and the relative abundance of the different taxa (Davenport et al., 2016). In line with this, no statistically significant association was found between microbiome composition and ethnicity, single-nucleotide polymorphisms, or overall genetic similarity in another study carried out in adults (Rothschild et al., 2018); similarly, the significant differences observed in early life between infants from three ethnic groups living in Singapore were no longer detectable at the age of 2 years (Xu et al., 2020). This latter study suggests that the environment is more influential than the host genetics.

Studies on animals (mice in particular), however, have demonstrated differences in the microbiome depending on the strain and after knock-out (KO) of specific genes (for reviews, see Spor et al., 2011; Campbell et al., 2012; Bubier et al., 2021). Some of these studies have nevertheless shown strong interaction between the effects of genetics and diet (Hildebrandt et al., 2009; Kashyap et al., 2013; Parks et al., 2013), which could explain the apparent contradictions in human studies.

## Conclusion

As noted in the above review of works on the lasting effects of neonatal changes in early microbiota, the effects of a modulator can range from altering the overall structure of the microbiota to altering a single bacterial population, so it is not so simple to determine whether programming exists or not. Current methods used to analyze the microbiota generate multiple indicators (alpha-diversity indices, global structure markers, absolute or relative abundance for different taxonomic levels), which raises the question of how many disparities make a “difference” and suggests a need to determine the criteria for defining what constitutes microbiome programming. It is unfortunate that the use of clustering in enterotypes, which condense information (Costea et al., 2018), remains limited in studies of infant microbiota, particularly studies about the lasting repercussions of neonatal modulators. A study of this type, however, suggests that clustering into enterotypes is unsuitable for studying the impact of perinatal factors, probably due to its extreme sensitivity to developmental stage (Xiao et al., 2021).

This review argues in favor of the absence of any long-term repercussions of early modulation of the human microbiome assembly on its adult composition. The advancement in age of the various cohorts constituted in the last decade (e.g., KOALA cf. Penders et al., 2006; CHILD study cf. Tun et al., 2017; TEDDY cf. Stewart et al., 2018) should facilitate confirmation of this assessment over longer periods, subject to resampling the individuals concerned with a view of characterizing their microbiome. Our conclusion is otherwise consistent with the findings from large Flemish-Belgian and Dutch cohorts, where no association was found between variations in microbiome composition and early life events such as mode of birth and infant nutrition (Falony et al., 2016). It is also in line with recently identified major lifestyle impacts on adult gut microbiota relating to both settlement (Jha et al., 2018) and international migration (Vangay et al., 2018), with variations across the groups studied only partly explained by differences in diet. Altogether, these findings suggest that the pioneer bacteria that colonize the gut probably have less influence than originally thought.

The absence of any enduring impact on the intestinal microbiota by neonatal modulators rules out the possibility of the microbiota having any role in the DOHaD as a “memory” of the first 1,000 days, but this does not exclude the microbiota potentially acting as a mechanistic relay of the early modulations to which it was subject in the neonatal period. In this regard, various epidemiological studies have established statistical associations between early alteration of the intestinal microbiota and the subsequent occurrence of particular pathophysiologies (reviewed by Blottière et al., 2013; Tamburini et al., 2016; Milani et al., 2017; Stinson, 2020). It has been documented in particular in the case of birth by CS (Korpela, 2021; Ríos-Covian et al., 2021) and perinatal antibiotic therapies (Azad et al., 2017; Aires, 2021), but these associations should be viewed with caution due to the possibility of biases relating to reverse causalities and the fact that the repercussions of CS are not limited to modulation of the microbiota (King, 2021). Interventional studies on animal models are more convincing in this respect; to date, several studies have demonstrated associations between early microbiotal perturbations and subsequent physiological consequences (e.g., Cox et al., 2014 or Pocheron et al., 2021), demonstrating the need to consider early microbiota alterations in the DOHAD process.

## Author Contributions

CM made the plan and performed the manuscript writing. CM and HB developed the content of the review according to their discussions. Both authors revised and approved the manuscript.

## Conflict of Interest

The authors declare that the research was conducted in the absence of any commercial or financial relationships that could be construed as a potential conflict of interest.

## Publisher’s Note

All claims expressed in this article are solely those of the authors and do not necessarily represent those of their affiliated organizations, or those of the publisher, the editors and the reviewers. Any product that may be evaluated in this article, or claim that may be made by its manufacturer, is not guaranteed or endorsed by the publisher.
